# Ruptured sinus of Valsalva aneurysm developed forty years after aortic valve replacement by Starr-Edwards mechanical valve

**DOI:** 10.11604/pamj.2015.20.80.5983

**Published:** 2015-01-29

**Authors:** Hicham Labsaili, Pascal Leprince

**Affiliations:** 1 Assistance Publique-Hôpitaux de Paris, Department of Cardiac and Thoracic Surgery, Institute of cardiology, La Pitié-Salpêtrière hospital, Paris, France

**Keywords:** Aneurysm, sinus of Valsalva, redo cardiac surgery

## Image in medicine

Sinus of Valsalva aneurysm is a rare anomaly, most often caused by a congenital absence of muscular and elastic tissue in the aortic wall of the sinus of Valsalva. It was first described in 1938. The incidence is higher in eastern than western populations. Various surgical repair techniques have been reported with good results. In this case, a 61 years old man, with history of aortic valve replacement by mechanical Starr-Edwards valve forty years ago, developed an aneurysm of sinus of Valsalva. After femoro femoral cardiopulmonary bypass and sternotomy, we found a rupture in non coronary sinus, the mechanical valve is removed and a biological Bentall procedure is performed.

**Figure 1:**
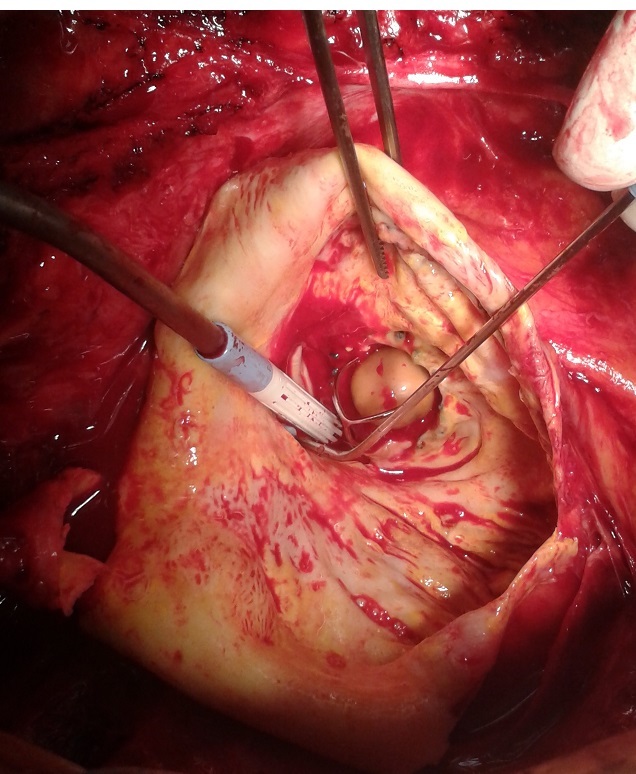
Opening of the aneurysm of the sinus of Valsalva and starting to remove the Starr-Edwards aortic valve, then a biological Bentall procedure was performed

